# Functional connectivity and gray matter deficits within the auditory attention circuit in first-episode psychosis

**DOI:** 10.3389/fpsyt.2023.1114703

**Published:** 2023-02-13

**Authors:** Mark T. Curtis, Alfredo L. Sklar, Brian A. Coffman, Dean F. Salisbury

**Affiliations:** Clinical Neurophysiology Research Laboratory, Department of Psychiatry, Western Psychiatric Hospital, University of Pittsburgh School of Medicine, Pittsburgh, PA, United States

**Keywords:** selective attention, first episode psychosis, phase-locking value, functional connectivity, phase-amplitude coupling, gray matter

## Abstract

**Background:**

Selective attention deficits in first episode of psychosis (FEP) can be indexed by impaired attentional modulation of auditory M100. It is unknown if the pathophysiology underlying this deficit is restricted to auditory cortex or involves a distributed attention network. We examined the auditory attention network in FEP.

**Methods:**

MEG was recorded from 27 FEP and 31 matched healthy controls (HC) while alternately ignoring or attending tones. A whole-brain analysis of MEG source activity during auditory M100 identified non-auditory areas with increased activity. Time-frequency activity and phase-amplitude coupling were examined in auditory cortex to identify the attentional executive carrier frequency. Attention networks were defined by phase-locking at the carrier frequency. Spectral and gray matter deficits in the identified circuits were examined in FEP.

**Results:**

Attention-related activity was identified in prefrontal and parietal regions, markedly in precuneus. Theta power and phase coupling to gamma amplitude increased with attention in left primary auditory cortex. Two unilateral attention networks were identified with precuneus seeds in HC. Network synchrony was impaired in FEP. Gray matter thickness was reduced within the left hemisphere network in FEP but did not correlate with synchrony.

**Conclusion:**

Several extra-auditory attention areas with attention-related activity were identified. Theta was the carrier frequency for attentional modulation in auditory cortex. Left and right hemisphere attention networks were identified, with bilateral functional deficits and left hemisphere structural deficits, though FEP showed intact auditory cortex theta phase-gamma amplitude coupling. These novel findings indicate attention-related circuitopathy early in psychosis potentially amenable to future non-invasive interventions.

## Introduction

Selective attention deficits are present before psychosis emerges, endure throughout illness, and are associated with poorer functional outcomes (1–4). The EEG-measured auditory event-related potential (or MEG-measured event-related field) ~100 ms post-stimulus (N100/M100) is enhanced with attention in healthy individuals but not in schizophrenia or first episode psychosis (FEP) (5–8). We recently showed that FEP have reduced M100 modulation by attention in primary auditory cortex, lateral belt, and parabelt (9). However, it is unclear if the pathology underlying this impairment is restricted to mechanisms within auditory sensory cortex or involves non-auditory executive control regions as well.

The attention-related cortical network includes prefrontal and posterior parietal cortices (10–12). FMRI-based work suggests a fronto-parietal system (including prefrontal cortex, posterior parietal cortex, and precuneus) actively modulates task-relevant information and a cingulo-opercular system (including anterior prefrontal cortex, frontal operculum, and anterior cingulate) maintains sustained attention (13, 14). The frontal and parietal areas involved in attentional control show slight localization differences for auditory and visual attention. The visual attention system engages the superior precentral sulcus and inferior precentral sulcus along with the intraparietal sulcus and visual cortex. The auditory-biased network involves the transverse gyrus, precentral gyrus, caudal inferior frontal cortex, superior temporal sulcus and gyrus, and auditory cortex (15, 16). Thus, these models agree that executive control of attention involves frontal regions communicating with parietal (medial or lateral) and enhancing activity in sensory cortices.

While fMRI provides high spatial resolution, it indirectly measures neural activity and is less sensitive to rapid changes <1 s. EEG and MEG directly measure neural activity, specifically synchronized postsynaptic currents, from groups of cortical neurons with high temporal millisecond resolution, necessary for examining sub-second sensory processes involved in N100/M100. Further, MEG provides higher spatial resolution compared to EEG because magnetic fields are unaffected by tissue boundaries (17).

In MEG and EEG, coordinated local processing and network communication are thought to be reflected by synchrony in neural oscillations. In humans and animals, attentional modulation of sensory activity is associated with increased gamma synchrony in sensory areas, which correlates with gamma synchrony in prefrontal cortex (PFC) (11, 18–21). Attention also influences sensory cortex coupling between low-frequency (4–13 Hz) phase and high-frequency power, termed phase-amplitude coupling (PAC). For example, alpha phase in infragranular layers of visual cortex entrains gamma amplitude in superficial layers (22–26). Communication between cortical areas occurs through coherence of the low-frequency oscillations, and this synchrony increases with attention (27–30). Visual attention modulates alpha connectivity between visual, parietal, and frontal regions (31, 32), while auditory attention changes theta connectivity (25, 33, 34).

Local sensory oscillations and long-range oscillatory connectivity deficits are present in psychosis and are related to cognitive impairment (35). In auditory cortex, gamma-band activity is impaired in early psychosis (36, 37). Cognitive control-related increase in PFC gamma activity is impaired in schizophrenia (38, 39). However, not all local oscillatory mechanisms are impaired, as PAC may be intact in schizophrenia (26, 40, 41). Distributed network connectivity is also altered in schizophrenia. Resting-state alpha activity is dysfunctional within several networks as early as the first-episode of schizophrenia (42). Oscillatory connectivity deficits are also associated with cognitive impairment in psychosis (43), and one study suggests deficits in attention-related alpha desynchronization are present even prior to psychosis onset (44). However, there has been relatively little additional study of oscillatory mechanisms associated with attentional impairments in early psychosis.

In addition to functional deficits, psychosis is associated with gray matter deficits (45–53), some present even before first psychosis. FEP show regional gray matter loss in frontal and temporal cortices (54–60). Gray matter in temporal and frontal lobes is related to cognitive functioning in chronic schizophrenia (50, 61, 62) and is associated with functional deficits, such as reduced mismatch negativity (63–66). Any functional deficits in local and long-range neural oscillations involved with failure of attentional modulation in FEP may be related to structural changes occurring at the same time.

The current study is a follow-up to our recent report that identified M100 attention modulation deficits in auditory cortex in FEP (9). To investigate functional and structural deficits in local and long-range networks underlying the M100 attentional modulation deficit in FEP, we used a multi-stepped strategy. In Analysis 1, whole-brain MEG broadband source activity during the M100 interval was examined to detect extra-auditory areas similarly activated with attention as sensory cortex. We hypothesized canonical attention-related regions, such as the prefrontal and posterior parietal cortices would increase activity with attention. To determine whether these areas were functionally connected to auditory cortex in the service of attention, we turned to the spectral domain of the MEG signal. In Analysis 2, a time-frequency analysis was conducted, and PAC between low-frequency phase and high-frequency gamma amplitude in primary auditory cortex were investigated to identify the carrier frequency driving the increased local sensory gamma activity during the M100, putatively sent from executive areas. We hypothesized that attention would increase coupling between gamma amplitude and theta or alpha low frequency phase. In Analysis 3, attention networks were defined by synchrony at the identified carrier frequency between regions. Precuneus subregions were used as seed regions due to their identification in Analysis1 (see below) and their known role in attention (14). Spectral connectivity deficits within this network were then investigated in FEP. We hypothesized that there would be a cortical network with increased low frequency connectivity with attention, and connectivity deficits within this network would be present in FEP. In Analysis 4, gray matter deficits within the attention network were investigated in FEP, and we hypothesized gray matter within this network would be related to functional attention-related deficits in FEP.

## General methods and materials

### Participants

This study included the same participants as Curtis and colleagues (9), comprising 27 FEP (recruited from Western Psychiatric Hospital with <2 months lifetime antipsychotic exposure) and 31 healthy controls (HC). No participant had a history of concussion or head injury with sequelae, history of alcohol or drug addiction or detox in the last 5 years, or neurological comorbidity. Groups were matched for age, gender, parental social economic status, and premorbid IQ, estimated by the Wechsler Abbreviated Scale of Intelligence (WASI) vocabulary t-score (Table 1). Participants had healthy hearing confirmed with audiometry. Participants provided informed consent and were paid for participation. The work was carried out in accordance with the Declaration of Helsinki.

**Table 1:** Demographic, neuropsychological, and clinical information (previously reported in Curtis et al., 2022).

	Mean ± SD		*t/X^2^*	*p*	Cohen’s *d*
	HC	FEP			
Sociodemographic data					
Age (years)	24.9 ± 5.7	23.4 ± 4.5	1.12	0.27	0.29
Gender (M/F)	22/9	17/10	0.42	0.52	
SES	41.8 ± 13.1	29.8 ± 13.9	3.39	<0.01	0.89
Parental SES	48.5 ± 10.9	43.3 ± 13.4	1.64	0.11	0.43
Neuropsychological tests					
WASI Vocab T-Score	52.7 ± 6.2	49.2 ± 9.6	1.67	0.11	0.43
MCCB-total	50.7 ± 7.2	30.8 ± 14.6	6.71	<0.01	1.73
Symptoms					
PANSS Total		76.7 ± 19.8			
PANSS Positive		20.0 ± 6.5			
PANSS Negative		17.9 ± 6.0			
PANSS General		38.9 ± 9.8			
SAPS Global		6.2 ± 3.7			
SANS Global		10.1 ± 4.1			
Medication data					
Medication		242.3 ± 139.5			

FEP and HC groups were matched for age, gender, PSES, and WASI Vocab T-Score (mean ± SD). SES, hollingshead index of socioeconomic status. WASI, Wechsler abbreviated scale of intelligence. MCCB, MATRICS consensus cognitive battery composite scaled t score. PANSS, positive and negative syndrome scale. SAPS, scale for the assessment of positive symptoms. SANS, scale for the assessment of negative symptoms. Medication, chlorpromazine equivalents.

FEP diagnoses were based on the Structured Clinical Interview for DSM-IV (SCID-P) (67). Provisional diagnoses at baseline were confirmed 5–7 months later. Symptoms were rated using the using the Positive and Negative Syndrome Scale (PANSS) (Table 1) (68). Cognitive ability on tests sensitive to psychosis was assessed with the MATRICS Consensus Cognitive Battery (69).

Of the 27 FEP participants,17 received a diagnosis of schizophrenia (paranoid: *n* = 7; undifferentiated: *n* = 10), 1 of schizoaffective disorder (bipolar subtype), 2 of schizophreniform disorder, and 2 of psychotic disorder NOS. Five individuals received affective disorder diagnoses (Bipolar I disorder = 4; Major depressive disorder = 1), all with psychotic features. The majority of the FEP participants were medicated (21/27, 78%). Due to the differences in clinical manifestations between schizophrenia and other diagnoses for FEP, preliminary analyses investigating potential differences between FEP with a schizophrenia diagnosis and FEP without a schizophrenia diagnosis are reported in the Supplementary materials (Supplementary Tables S1, S2).

### Task

An oddball task with 340 standard tones (1 kHz, 50 ms duration, 10 ms rise/fall) and 60 deviant tones (1.2 kHz, 50 ms duration, 10 ms rise/fall) with stimulus onset asynchrony of 1,050–1,550 ms was presented twice. In one condition, participants ignored tones and attended a silent video. In the second condition, participants ignored the silent video and attended tones, pressing a button to deviant tones. Blocks were counterbalanced. Responses to standard tones, where M100 enhancement *via* attention is observed, were analyzed. To demonstrate participants were attending, hit rate to the target tones and false alarms were calculated. HC were more accurate (90.5 ± 12.6%) than FEP (77.6 ± 23.7%, *p* = 0.02), while groups did not differ on false alarms (HC: 2.2 ± 7.3, FEP: 3.6 ± 5.7, *p* = 0.44). This performance with relatively low false alarms demonstrates that all individuals were attempting to attend to the tones.

### MEG data acquisition and processing

MEG data were recorded in a magnetically shielded room with a 306-channel whole-head system (Elekta Neuromag), consisting of 102 triplets (1 magnetometer and 2 planar gradiometers) at 1000 Hz digitization with a bandpass filter of 0.1–330 Hz. Eye blinks and movements were recorded with bipolar leads placed above and below the left eye and lateral to the outer canthi of both eyes. Cardiac activity was recorded with bipolar ECG leads. A 3D-digitizer (ISOTRAK; Polhemus, Inc., Colchester, VT) was used to continuously record the location of 4 head position indicator coils. Neuromag MaxFilter software^1^ was used to correct for head motion. Temporal signal space separation was used to remove electromagnetic noise originating from outside the MEG helmet (70). Channels and segments of data with excessive noise *via* visual inspection were removed with EEGLAB (71). A high-pass filter (0.5 Hz; 12 dB/oct) was applied to the data, and an adaptive mixture independent component analysis was performed to remove eye-blink and ECG components. Any removed channels were then interpolated with spherical channel interpretation in EEGLAB.

MEG processing was performed with Brainstorm (72). A low-pass (20 Hz) filter was applied to remove muscle and other high-frequency artifacts. Trials were segmented from 100 ms before to 1,000 ms after stimulus onset. The average baseline voltage was subtracted, and trials that exceeded ±5pT were rejected.

For source activity, MEG sensor data was registered to each participant’s structural MRI (see MRI methods). Sources were constrained to the individual’s cortical surface. The forward solution was modeled as overlapping spheres, and a noise covariance matrix was calculated from the baseline window of all trials. Source activity was estimated using minimum norm estimation with a dipole constraint of 0.4 and depth weighting applied. Current density values were normalized with dynamic statistical parametric maps based on the variance in the prestimulus baseline.

### MRI data acquisition and processing

MRI acquisition and processing steps were the same as Curtis and colleagues (9). The following data were acquired on a Siemens 3T MAGNETOM Prisma scanner using a 32-channel phase array head coil: A single-band reference image with no slice acceleration at the beginning of each run; T1-weighted 3D MPRAGE images (TR/TE/TI = 2400/2.22/1000 ms, flip angle = 7°, FOV = 256 × 240mm, voxel size = 0.8 mm^3^, 208 slices, GRAPPA acceleration factor = 2); T2-SPACE images (TR = 3,200 ms TE = 563 ms, FOV = 256 × 240, voxel size = 0.8 mm^3^, 208 slices); A fieldmap (TR = 731 ms, TE = 4.92/7.38 ms, FOV = 208 × 180, voxel size = 2.0 mm^3^ voxel size, 72 slices); Ten minutes eyes-open, resting BOLD fMRI multiband data (TR = 800 ms, TE = 37 ms, multiband factor = 8, flip angle = 52°, FOV = 208 × 208mm, voxel size = 2.0 mm^3^, 72 slices); and Spin echo EPI images with reversed phase encoding directions (TR = 8,000 ms, TE = 66 ms, flip angle = 90°, FOV = 208 × 208mm, voxel size = 2.0 mm^3^, 72 slices).

The HCP-pipelines^2^ were used for MRI processing (73). Briefly, structural images were corrected for gradient nonlinearity, readout, and bias field, followed by AC-PC alignment. Myelin maps were created by the T1w/T2w ratio. White and pial surfaces were generated with Freesurfer (v6.0), refined using T2w data, and registered with a multimodal surface matching algorithm (MSMsulc) to the Conte69 template (74, 75).

FMRI data were collected with the structural MRI and processed with the HCP pipelines. The fMRI and myelin data were not analyzed here but were utilized for the multimodal surface matching algorithm (MSMall) algorithm implemented in the HCP pipelines to improve registration, alignment between participants, and utilize the HCP parcellation. FMRI data were processed with the HCP pipelines. FMRI data were corrected for gradient-nonlinearity. A 6 DOF FLIRT registration to the single-band reference image was used for motion correction. The spin-echo images were used to correct functional distortion. The single band reference image was registered to the T1w image with FreeSurfer’s BBRegister (76). The data were brain masked and intensity normalized (whole-brain mean = 10,000). Volumetric fMRI data were sampled to the individual’s native surfaces and resampled to a standard 32 k fs_LR surface. The ICA + FIX pipeline was used to remove artifactual noise (77, 78). Individuals were registered to a group average atlas surface using a two-stage MSMall algorithm, utilizing resting-state fMRI, cortical folding, and myelination information (74). The group average HCP-Multimodal Parcellation was applied to individuals’ cortical surface in the HCP connectome workbench and resampled to fsaverage space.^3^ In Freesurfer, this file was resampled to the individual’s surface and imported into Brainstorm.

## Analysis 1

### Methods and materials

#### Whole brain M100 window broadband source analysis

To investigate healthy attention modulation, data from HC were analyzed over the 80–140 ms post-stimulus, when M100 enhancement was previously observed in auditory cortex (9). Cortical source activity was calculated for each vertex for the attend and ignore conditions. Statistical *t*-tests were calculated for differences between conditions for each vertex. Permutation testing (5,000 permutations) was used for statistical significance with FDR correction for multiple comparisons (*q* < 0.05).

### Results

#### Whole brain M100 window broadband source analysis

**Figure 1:**
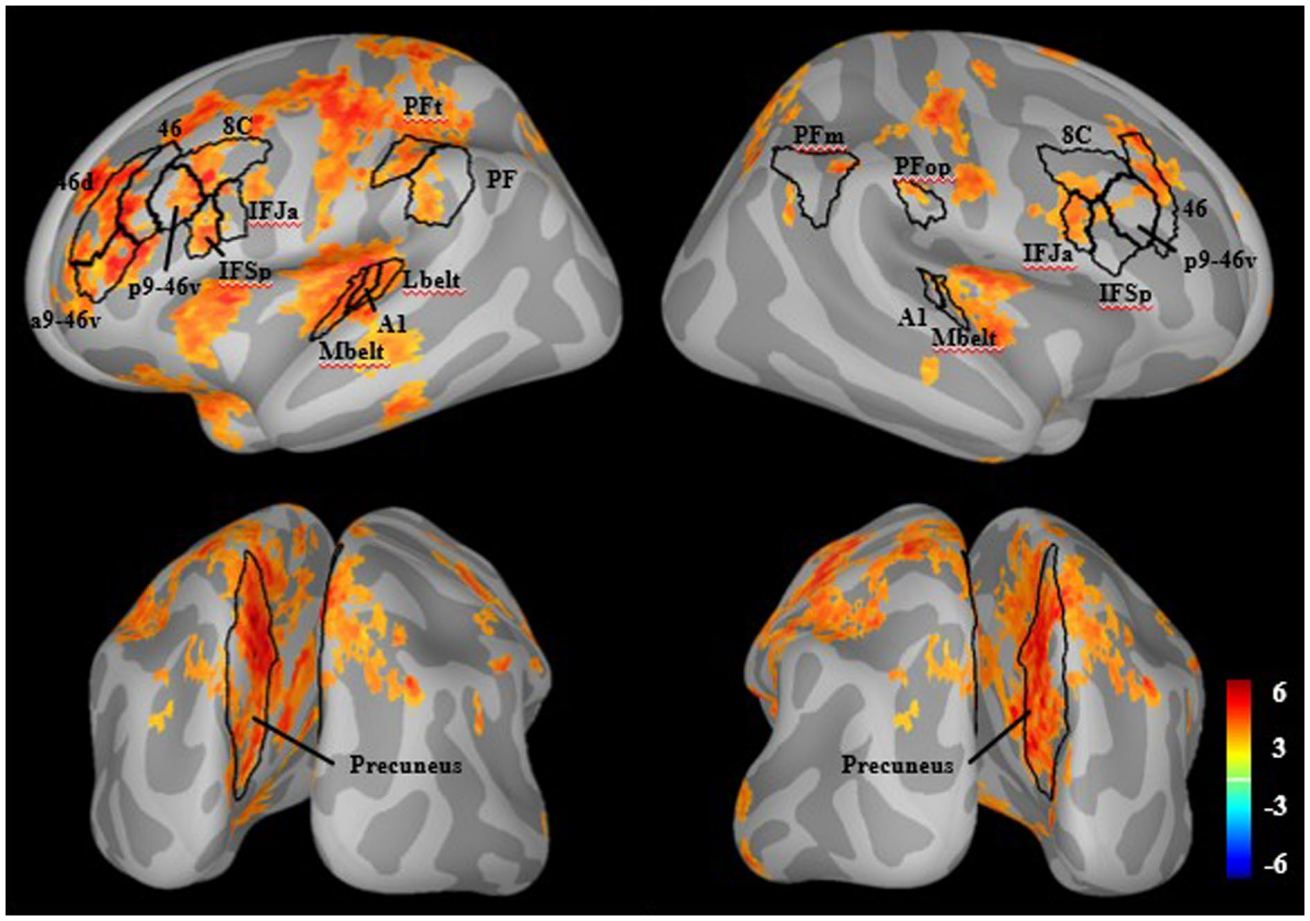
Whole-brain M100 modulation in healthy individuals. There were significant increases in source-resolved MEG activity (dspm) with attention compared to ignore in healthy individuals (*q* < 0.05). Outlined in black are regions aligning with the general hypothesized network including prefrontal, posterior parietal, precuneus, and auditory cortices.

In HC, there were significant increases with attention in many areas associated with canonical executive attention networks, including bilateral dorsolateral prefrontal cortex (8C, 46, p9-46v), inferior frontal cortex (IFJa, IFSp), lateral parietal cortex (left PF, left PFt, right PFop, right PFm), precuneus, and auditory cortex (A1, MBelt). There was also significant activity in left superior parietal, middle temporal, insular, cingulate, and somatosensory areas (Figure 1).

### Brief discussion

The whole brain analysis of source activity during the M100 time-window revealed increased activity in canonical executive attention network areas (14, 16). There was particularly strong involvement of the bilateral precuneus. The precuneus, a core component of the default mode network, is also a hub for attention, as it has a central role in changing attentional states during various cognitive tasks, including auditory attention (79–82).

This analysis demonstrates activity increases with attention in extra-auditory cortical areas during the M100 but does not determine any direct role in auditory attention enhancement. Hence, there was a need to examine dynamics between areas, which was accomplished with functional connectivity in the spectral domain. The primary goal of analysis 2 is to identify the carrier frequency for subsequent connectivity analyses. To accomplish this, a time-frequency analysis in primary auditory cortex was performed and coupling between executive low-frequency carrier phase and local sensory high-frequency gamma amplitude was investigated in auditory cortex regions of interest (ROIs).

## Analysis 2

### Methods and materials

#### Time-frequency analysis

The processing steps were the same with the omission of the low-pass (20 Hz) filter. In Brainstorm, the time-frequency decomposition of broadband source data from each trial was calculated with Morlet wavelets for 40 bins logarithmically spaced between 4 and 100 Hz (central frequency: 1 Hz, FWHM: 3 s). Trials were normalized to event-related perturbation during a 600 ms pre-stimulus baseline period, to investigate low frequencies, and averaged for each individual.

#### Phase-amplitude coupling

Phase-amplitude coupling (PAC) was calculated with the Ozkurt and Schnitzler method (83). All trials (0–1,000 ms post-stimulus) were concatenated, and Morlet wavelets were used for low-frequency (4–13 Hz) phase and high-frequency amplitude (30–100 Hz). PAC was calculated at each vertex of the ROI and averaged within the ROI. This was done in auditory ROIs (bilateral A1, LBelt, and PBelt).

#### Statistical analysis

To gain power to detect the carrier frequency, all individuals were included in the initial between-task comparisons. Within each ROI, a cluster-based permutation test was used to calculate significant differences between conditions. Permutation testing was used for the PAC analysis and for the between-group comparisons. An exploratory analysis of all ROIs was performed to investigate correspondence in which low-frequency phase was involved in coupling. Uncorrected t-statistics are reported for the highest instance of PAC within each region.

### Results

#### Time-frequency

**Figure 2:**
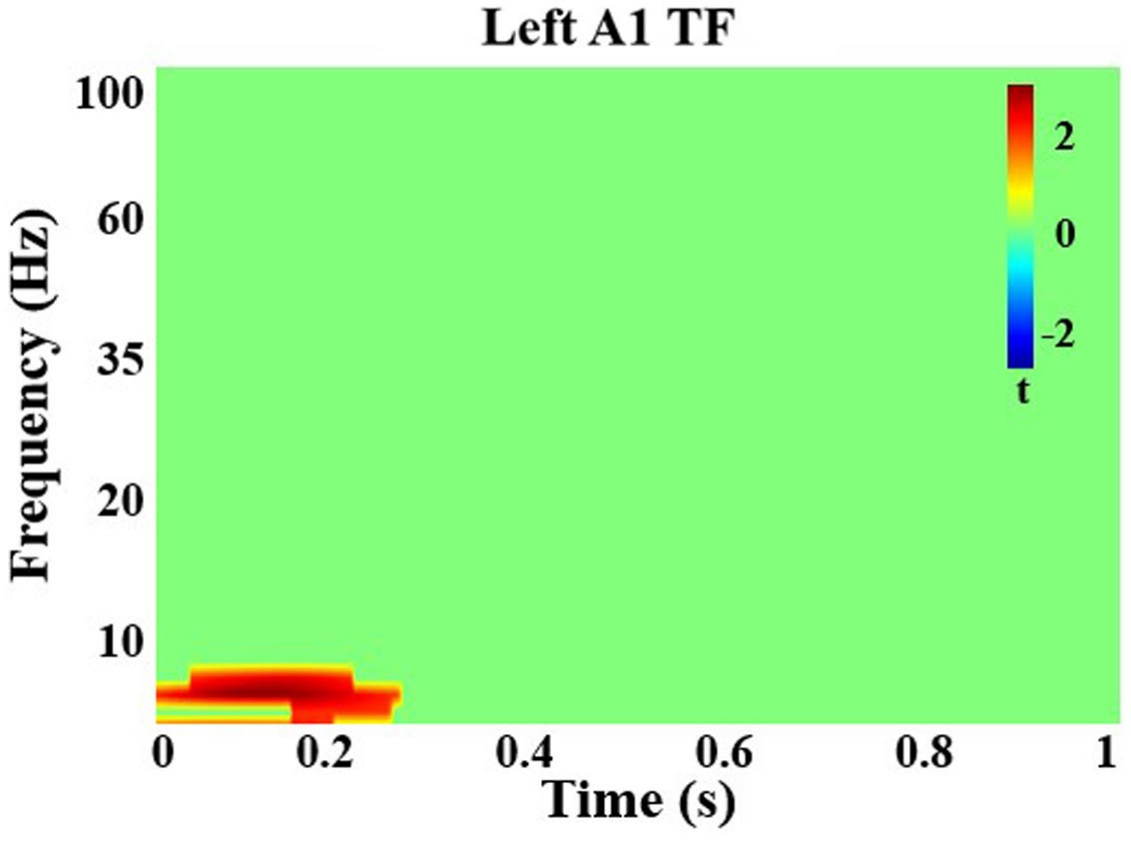
Time-frequency analysis in left primary auditory cortex. There was a significant increase in theta frequency power with attention in left auditory cortex from 0 to 330 ms in all individuals.

Among all participants, after correction for multiple comparisons, there was a significant increase in theta (5–7 Hz) power in left A1 with attention from 0 to 330 ms (*q* < 0.05; Figure 2). There were no significant group differences. In right A1, there were no significant differences with attention and no group differences.

#### PAC exploratory analysis

**Figure 3:**
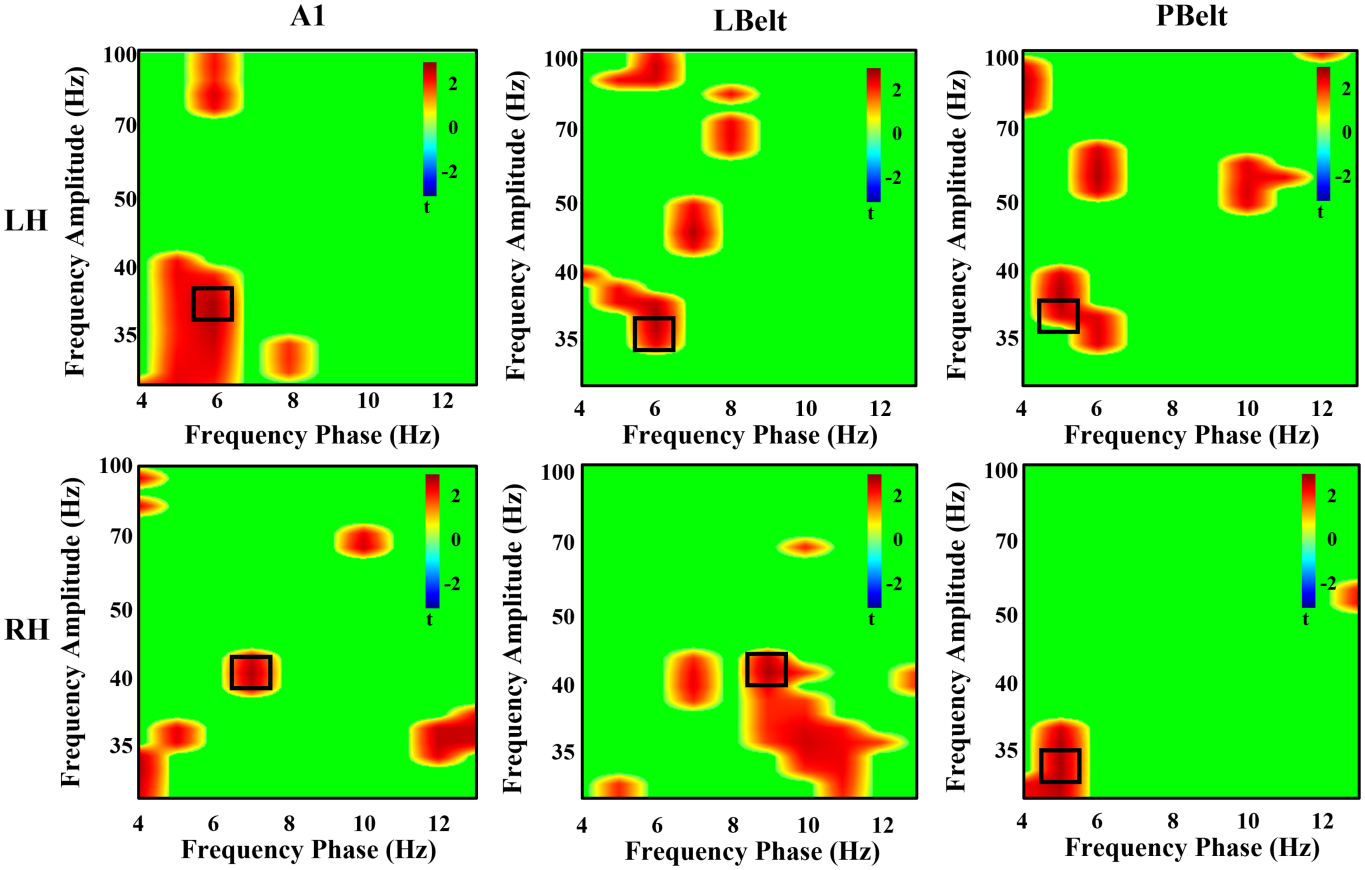
Exploratory uncorrected phase-amplitude coupling analysis in auditory cortex. There were uncorrected significant increases in coupling between low-frequency phase and gamma power. All, except right LBelt, areas showed the greatest coupling increase with attention between theta phase and gamma power. Right LBelt showed the greatest coupling increase with attention between alpha phase and gamma power. Peak coupling within each area is outlined in black.

In all participants, the highest instances of PAC increase in each region are reported in Table 2 and shown in Figure 3. All regions, except for right LBelt, had the greatest PAC between gamma amplitude and identified theta (5–7 Hz) phase. Right LBelt had the greatest PAC between gamma amplitude and alpha (8–10 Hz) phase.

**Table 2:** Phase-amplitude coupling in auditory cortex.

	Mean ± SD		*t*	*p*
	Attend (*n* = 58)	Ignore (*n* = 58)		
Left hemisphere				
A1	1.0×10^−5^ ± 4.1×10^−6^	8.3×10^−6^ ± 2.5×10^−6^	3.60	<0.001
LBelt	1.0×10^−5^ ± 4.0×10^−6^	8.7×10^−6^ ± 2.8×10^−6^	2.81	0.007
PBelt	1.2×10^−5^ ± 5.6×10^−6^	1.0×10^−5^ ± 4.1×10^−6^	2.53	0.014
Right hemisphere				
A1	9.6×10^−6^ ± 4.2×10^−6^	8.1×10^−6^ ± 3.6×10^−6^	2.73	0.008
LBelt	7.6×10^−6^ ± 3.3×10^−6^	6.2×10^−6^ ± 2.0×10^−6^	3.55	<0.001
PBelt	1.1×10^−5^ ± 4.0×10^−6^	9.6×10^−6^ ± 3.1×10^−6^	2.75	0.008

The instance of maximal coupling between low-frequency phase and high-frequency amplitude in all participants during attend and ignore conditions. All participants increased PAC with attention in all regions (except right LBelt) between theta phase and gamma amplitude. Participants increased PAC with attention between alpha phase and gamma amplitude in the right LBelt. A1, primary auditory cortex; LBelt, lateral belt; PBelt, parabelt.

#### PAC corrected statistics

**Figure 4:**
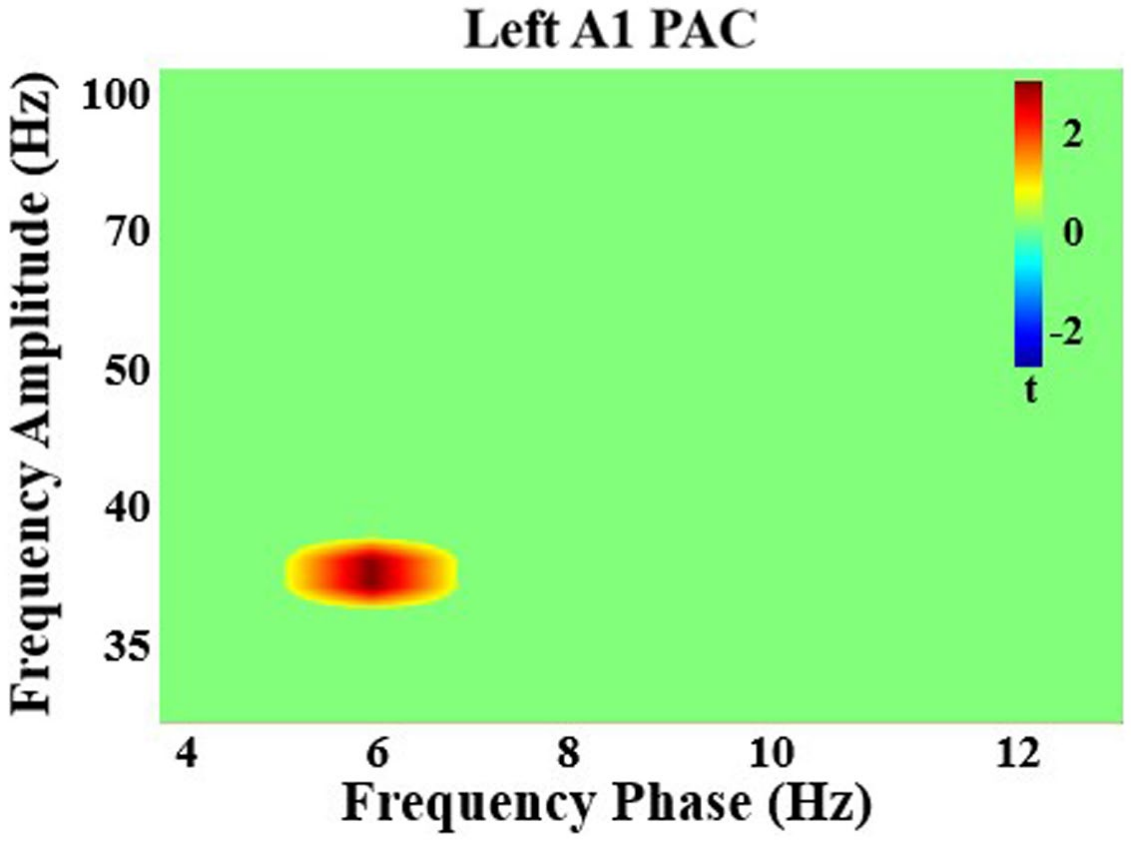
Significant phase-amplitude coupling analysis in left primary auditory cortex. There was a significant increase in coupling with attention between theta frequency phase and gamma frequency amplitude in left auditory cortex in all individuals.

After multiple comparisons correction, in all participants there was a significant increase in PAC with attention in left A1 between theta phase (5–7 Hz) and gamma amplitude (35–40 Hz; *q* < 0.05). There were no significant group differences (*q* > 0.05; Figure 4).

### Brief discussion

Within primary auditory cortex, theta power increased with attention from 0–330 ms, a time-window that includes the M100 (80–140 ms). In all areas, except right LBelt, theta phase was associated with local gamma synchrony, although multiple comparisons correction revealed significant effects only in left primary auditory core. This provides evidence that the low-frequency carrier frequency that modulates sensory regions with attention in this task is the theta frequency band. PAC in sensory cortex was intact in FEP, suggesting the mechanism underlying attention modulation deficits in early psychosis exists at the extra-auditory network level. To determine the nodes of the distributed attention network, the next analysis focused on attention modulation of whole-brain oscillatory network connectivity in theta band and potential functional connectivity deficits in FEP.

## Analysis 3

### Methods and materials

#### Functional connectivity

Phase locking value (PLV) was used to assess spectral synchrony and functional connectivity within the theta-band (5–7 Hz) (84, 85). Connectivity was assessed for all attend and ignore trials. Cortical source activity was band-pass filtered (5–7 Hz), and a Hilbert transform was used to obtain instantaneous phase. The PLV between two regions *i* and *j* was calculated:

$$PLV_{ij}=\frac{1}{T}\;\left|\overset{T}{\underset{t=1}{\sum }}e^{-i\left(\varphi i\left(t\right)-\varphi j\left(t\right)\right)}\right|$$

In the above equation, *T* is the number of data points in the time series and φ(t) represents the phase of each signal at timepoint *t*.

#### Precuneus clustering

With its central role in attention processing and involvement in the attention enhancement during the M100 window (Analysis 1), the precuneus was used as the seed region for functional connectivity. Noting the many roles of the precuneus, functional subsystems may exist. Thus, the precuneus was parcellated into 12 bilateral subregions with the HCP-MMP. PLV was calculated between each vertex in each seed region and each vertex in every other cortical parcel and then averaged within each parcel. This was done in HC for attend and ignore conditions, and t-values comparing conditions were calculated for each subregion. To identify potential subsystems, subregions were clustered based on connectivity difference patterns with t-distributed stochastic neighbor embedding (t-SNE) implemented in Matlab and k-means clustering (86). The elbow method determined the optimal cluster number. Within-cluster sum of squared errors (WSS) was calculated, and cluster number was chosen when greater than 90% of the variance was explained.

Once clustered, the precuneus parcels were merged, and PLV connectivity was calculated again using the entire cluster as a seed region to determine which cortical parcels had significantly greater connectivity with attention.

#### Functional connectivity analysis

An FDR of 0.1 was used to control for multiple comparisons of the whole brain connectivity between precuneus clusters and HCP-parcellated regions in HC. An FDR of 0.1 was selected to balance the expected relatively small effect sizes due to relatively smaller sample sizes (31 HC) and large number of comparisons (~360). Further, the goal of this first analysis was to discover areas with strong connectivity changes in HC. Then, these discovered network regions were examined in FEP using repeated measures ANOVAs with one between-subjects factor (Group: Control or FEP) and one within-subject factor (Regions).

### Results

#### Precuneus subregion clustering

**Figure 5:**
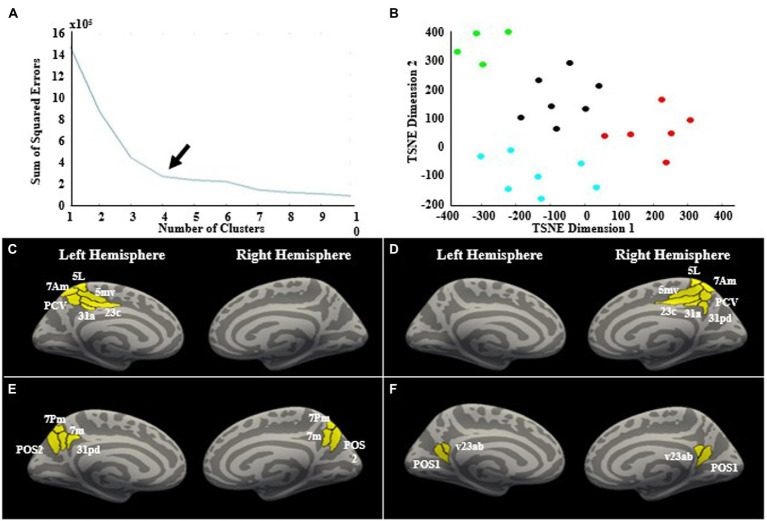
Cluster analysis. **(A)** K-means clustering and the elbow-method was used to determine the optimal number of clusters, which was determined to be four clusters, indicated by the arrow. **(B)** The two-dimensional t-SNE plot with each point representing a precuneus subregion. Each region’s location on the plot is determined by the region’s whole-brain connectivity profile in the theta band. If regions have more similar connectivity profiles, they will be located closer to each other on the t-SNE plot. The clusters are denoted by different colors. The four clusters are shown on the cortical surface. There was a left hemisphere cluster **(C)**, a right hemisphere cluster **(D)**, and two bilateral clusters **(E,F)**.

The clustering analysis yielded 4 subregions based on differential theta band whole brain connectivity (Figure 5). Cluster 1 included left hemisphere regions 23c, 31a, 5L, 5mv, 7am, and precuneus visual area (PCV). The second cluster included right hemisphere regions 23c, 31a, 31pd, 5L, 5mv, 7am, and PCV. The third cluster included left 31pd, left 7 m, left 7 pm, left posterior precuneus 2 (POS2), right 7m, right 7pm, and right POS2. The fourth cluster included left posterior precuneus 1 (POS1), left ventral 23ab, right POS1, and right ventral 23ab.

#### Connectivity changes with attention in HC

**Figure 6:**
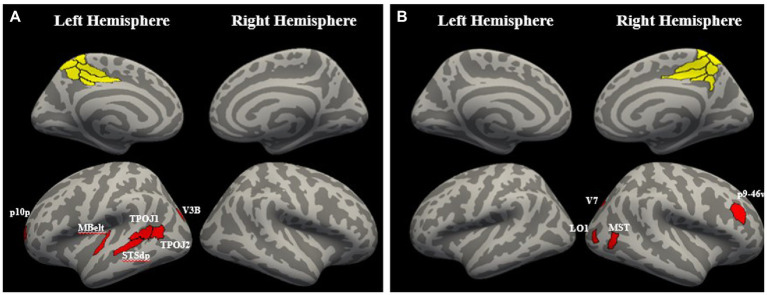
Regions with significant connectivity increases with attention. **(A)** The left hemisphere precuneus cluster had significant increases in theta phase connectivity with attention with left medial belt (MBelt), left dorsal posterior superior temporal sulcus (STSdp), left temporo-parieto-occipital junction 1 and 2 (TPOJ1 and TPOJ2), left visual area 3b (V3B), and posterior frontal pole area 10 (p10p; *q* < 0.1). **(B)** The right hemisphere precuneus cluster had significant increases in theta phase connectivity with attention with right medial superior temporal area (MST), right lateral occipital area 1 (LO1), right visual area 7 (V7), right posterior area 9-ventral area 46 in the dorso-lateral prefrontal cortex (p9-46v; *q* < 0.1).

In HC, cluster 1 showed significantly greater connectivity with attention with left medial belt (MBelt), left dorsal posterior superior temporal sulcus (STSdp), left temporo-parieto-occipital junction 1 and 2 (TPOJ1 and TPOJ2), left visual area 3b (V3B), and posterior frontal pole area 10 (p10p; *q* < 0.1). The right lateralized cluster 2 showed significantly greater connectivity with attention with right medial superior temporal area (MST), right lateral occipital area 1 (LO1), right visual area 7 (V7), right posterior area 9-ventral area 46 in the dorsolateral prefrontal cortex (p9-46v; *q* < 0.1; Figure 6). The other two precuneus clusters did not have connectivity increases with attention that survived FDR correction (*q* > 0.1).

#### Connectivity group differences

**Figure 7:**
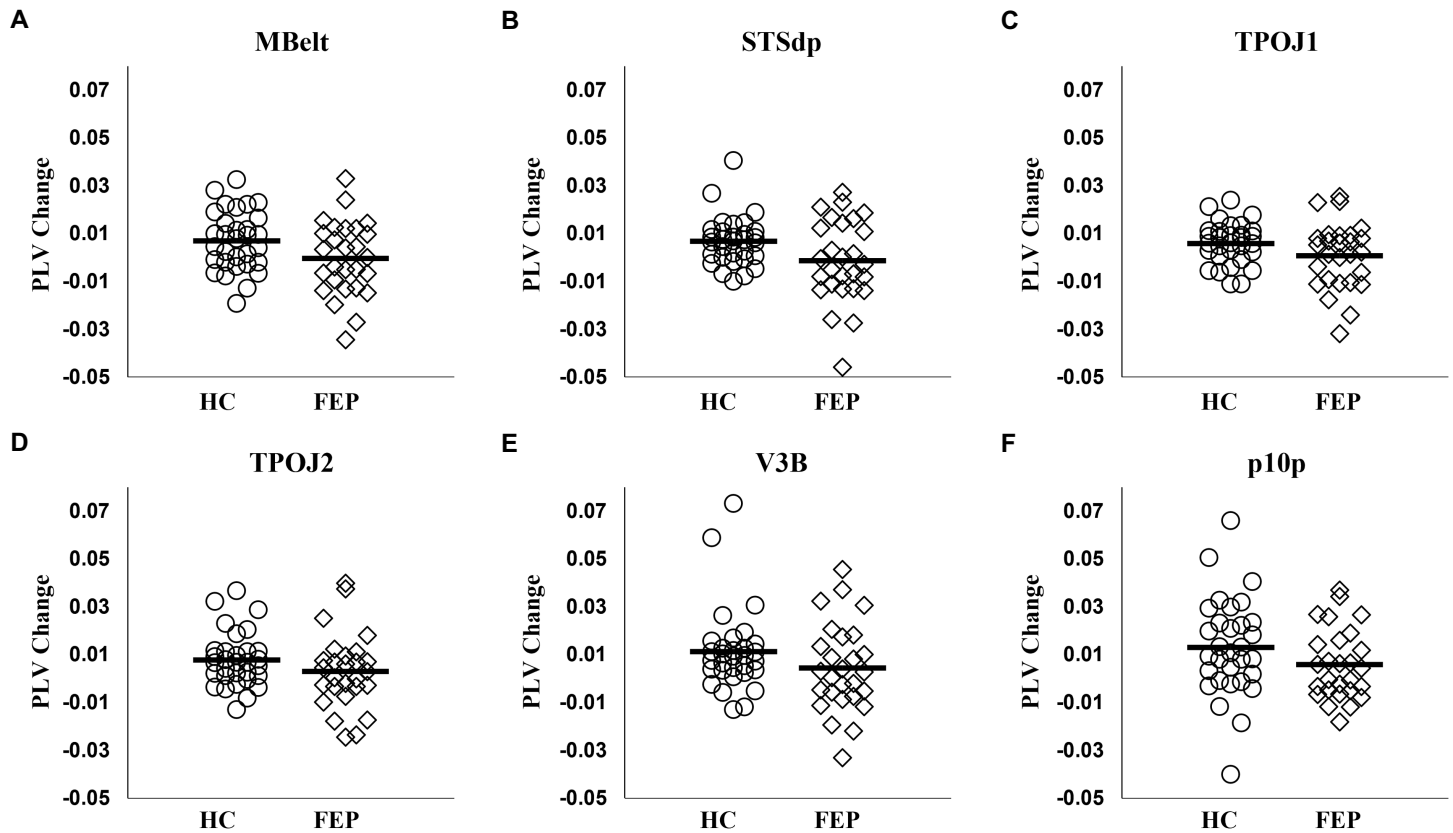
Left hemisphere network connectivity changes in HC and FEP. There was a significant reduction in the modulation of theta band connectivity with attention (measured with change in phase-locking value) across regions in the left hemisphere network in FEP **(A–F)**.

**Figure 8:**
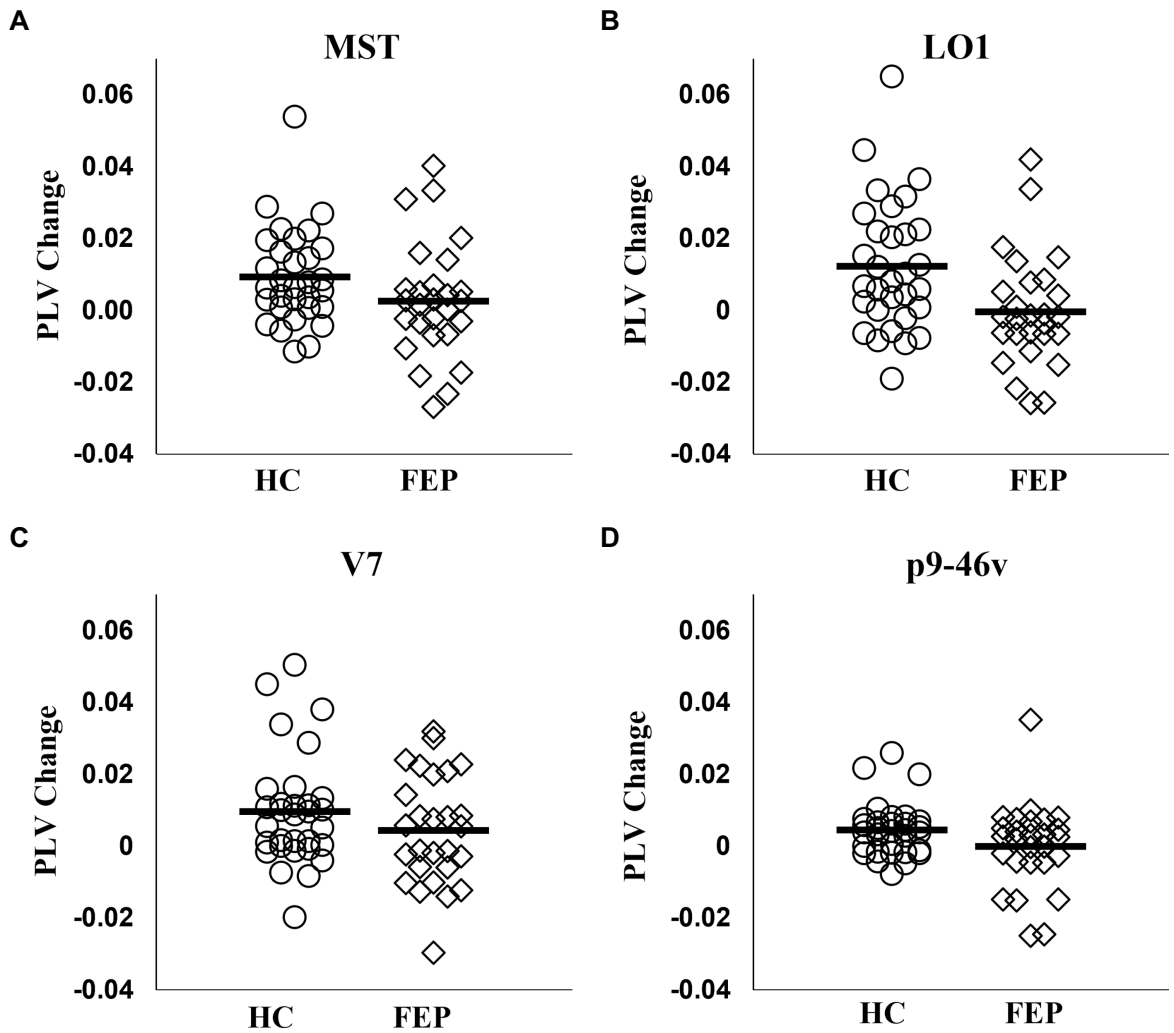
Right hemisphere network connectivity changes in HC and FEP. Similar to the left hemisphere network, there was a significant reduction in the modulation of theta band connectivity with attention (measured with change in phase-locking value) across regions in the right hemisphere network in FEP **(A–D)**.

FEP were significantly impaired in the ability to enhance connectivity with attention in both the left hemisphere (*F*_1,56_ = 8.58, *p* < 0.01; Figure 7; Table 3) and the right hemisphere (*F*_1,56_ = 7.67, *p* < 0.01; Figure 8; Table 3).

**Table 3:** Connectivity changes with attention.

	Mean ± SD		*t*	*p*
	HC (*n* = 31)	FEP (*n* = 27)		
Left hemisphere				
MBelt	0.0079 ± 0.012	−0.0013 ± 0.016	2.53	0.01
STSdp	0.0067 ± 0.010	−0.0014 ± 0.017	2.21	0.03
TPOJ1	0.0059 ± 0.008	0.0007 ± 0.014	1.72	0.09
TPOJ2	0.0078 ± 0.011	0.0030 ± 0.016	1.36	0.18
V3B	0.0112 ± 0.017	0.0044 ± 0.018	1.45	0.15
p10p	0.0131 ± 0.021	0.0059 ± 0.015	1.51	0.18
Right hemisphere				
MST	0.0092 ± 0.013	0.0026 ± 0.016	1.72	0.09
LO1	0.0123 ± 0.018	−0.0004 ± 0.016	2.82	0.01
V7	0.0096 ± 0.016	0.0044 ± 0.015	1.27	0.21
p9-46v	0.0045 ± 0.007	−0.0001 ± 0.011	1.78	0.08

Changes in theta band phase-locking value with the precuneus cluster with attention (Attend-Ignore) in healthy controls (HC) and individuals at the first episode of psychosis (FEP). MBelt, Medial belt; STSdp, dorsal posterior superior temporal sulcus; TPOJ1, temporo-parieto-occipital junction 1; TPOJ2, temporo-parieto-occipital junction 2; V3B, visual area 3b; p10p, posterior frontal pole area 10; MST, medial superior temporal area; LO1, lateral occipital area 1; V7, visual area 7; p9-46v, posterior area 9-ventral area 46.

### Brief discussion

Cluster analysis of whole brain synchrony using precuneus seed regions identified a left hemisphere and a right hemisphere network that showed increased theta synchrony with attention in HC. Importantly, FEP did not modulate theta synchrony within these networks. These novel findings indicate a distributed circuit involved in auditory attention modulation that is functionally impaired in early psychosis. The final analysis investigated if these functional measures, and corresponding deficits in FEP, were related to underlying gray matter.

## Analysis 4

### Methods and materials

#### Gray matter analysis

Gray matter volume and thickness differences were investigated in regions with significant functional connectivity enhancement from Analysis 3 (Left Hemisphere: MBelt, STSdp, TPOJ1, TPOJ2, V3B, and p10p: Right Hemisphere: MST, LO1, V7, and p9-46v). Analysis of covariance (ANCOVA) was performed on relative volumes [absolute volume/intracranial content (ICC)] and thickness with gender and age as covariates (The statistical conclusions remained the same when using ANCOVA on absolute volumes with intracranial content (ICC), gender, and age as covariates).

In HC and FEP, absolute volumes and thicknesses were correlated (Spearman’s rho) with PAC modulation in left A1, and network theta connectivity modulation. Correlations were also examined between gray matter in the left hemisphere network regions and symptom severity in FEP (PANSS Total, Positive, Negative, and General factors).

### Results

**Figure 9:**
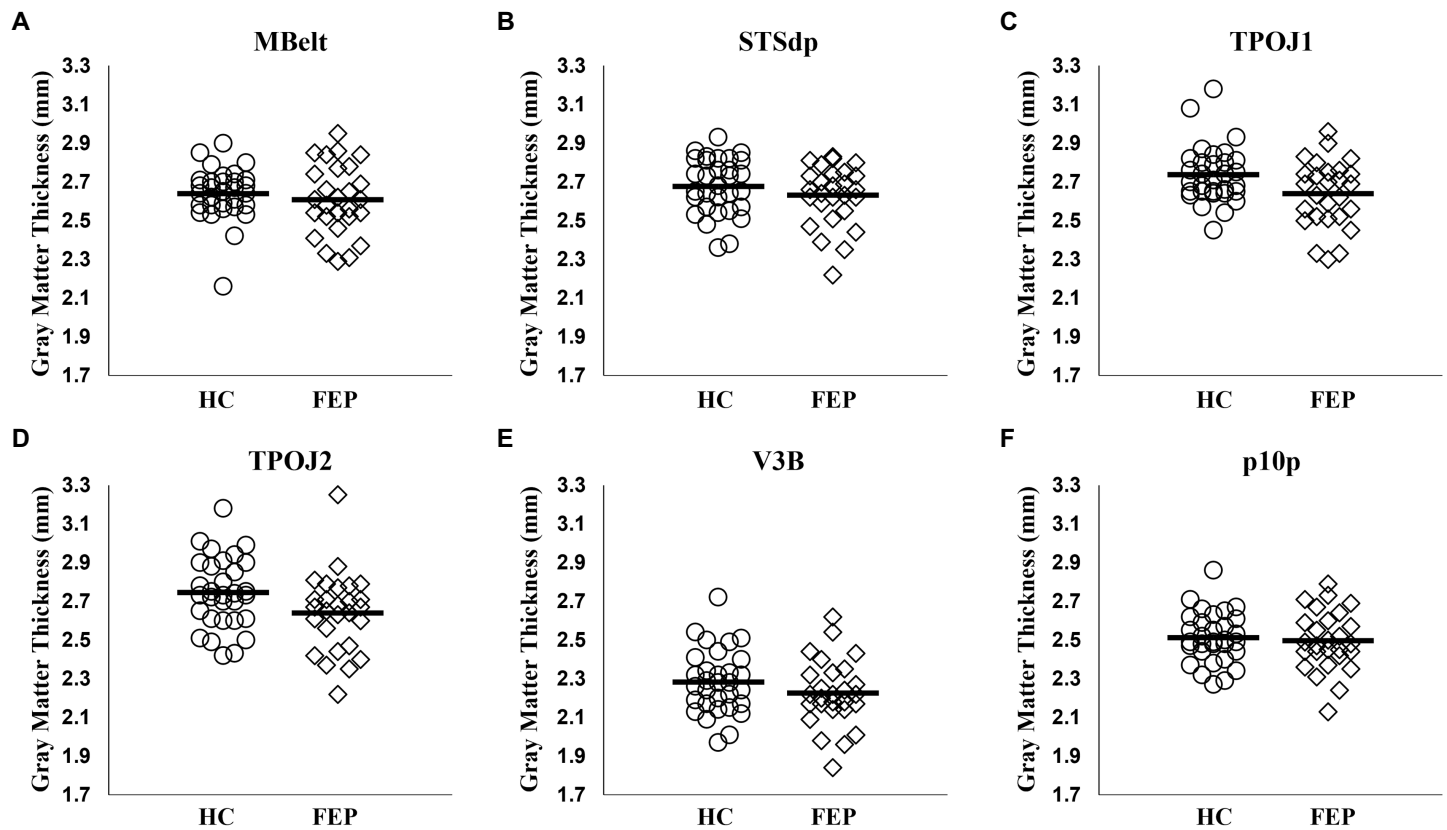
Gray matter thickness in the left hemisphere attention network. Compared to healthy controls, individuals with first-episode of psychosis (FEP) showed significant reductions in gray matter thickness across the regions in the left hemisphere precuneus attention network (*p* < 0.05; **A–F**).

In the left hemisphere network, FEP had significantly thinner gray matter (*F*_1,54_ = 5.14, *p* = 0.02; Figure 9). There were no differences in gray matter volume (*p* > 0.1). There were no group differences in gray matter volume or thickness within the right hemisphere network (*p*’s > 0.1). Thickness values are reported in Table 4, and volumes are reported in Table 5.

**Table 4:** Gray matter thickness.

	Mean ± SD		*t*	*p*
	HC (*n* = 31)	FEP (*n* = 27)		
Left hemisphere				
MBelt	2.64 ± 0.14	2.61 ± 0.18	0.78	0.44
STSdp	2.68 ± 0.14	2.63 ± 0.15	1.18	0.24
TPOJ1	2.74 ± 0.15	2.64 ± 0.17	2.38	0.02
TPOJ2	2.75 ± 0.18	2.64 ± 0.20	2.09	0.04
V3B	2.28 ± 0.17	2.22 ± 0.17	1.32	0.19
p10p	2.51 ± 0.13	2.50 ± 0.15	0.40	0.69
Right hemisphere				
MST	2.52 ± 0.18	2.49 ± 0.18	0.72	0.47
LO1	2.42 ± 0.20	2.45 ± 0.16	−0.80	0.43
V7	2.33 ± 0.20	2.25 ± 0.22	1.52	0.14
p9-46v	2.67 ± 0.15	2.72 ± 0.19	−1.12	0.27

Gray matter thickness in mm (mean ± SD) differences between healthy controls (HC) and individuals at the first-episode psychosis (FEP) from the ROIs in the left and right hemisphere attention networks. MBelt, Medial belt; STSdp, dorsal posterior superior temporal sulcus; TPOJ1, temporo-parieto-occipital junction 1; TPOJ2, temporo-parieto-occipital junction 2; V3B, visual area 3b; p10p, posterior frontal pole area 10; MST, medial superior temporal area; LO1, lateral occipital area 1; V7, visual area 7; p9-46v, posterior area 9-ventral area 46.

**Table 5:** Gray matter volume.

	Mean ± SD		*t*	*p*
	HC (*n* = 31)	FEP (*n* = 27)		
Intracranial content	1.20 × 10^6^ ± 0.15 × 10^6^	1.17 × 10^6^ ± 0.13 × 10^6^	0.74	0.46
Left hemisphere				
MBelt	0.065 ± 0.010	0.068 ± 0.011	−1.18	0.24
STSdp	0.103 ± 0.016	0.104 ± 0.014	−0.09	0.93
TPOJ1	0.103 ± 0.018	0.100 ± 0.020	0.55	0.59
TPOJ2	0.100 ± 0.030	0.101 ± 0.033	−0.11	0.91
V3B	0.039 ± 0.008	0.037 ± 0.010	0.65	0.52
p10p	0.110 ± 0.020	0.109 ± 0.190	0.10	0.92
Right hemisphere				
MST	0.050 ± 0.013	0.053 ± 0.014	−0.54	0.59
LO1	0.050 ± 0.012	0.050 ± 0.012	−0.04	0.97
V7	0.049 ± 0.013	0.047 ± 0.009	0.84	0.41
p9-46v	0.185 ± 0.053	0.190 ± 0.039	−0.41	0.68

Differences in gray matter volume corrected for intracranial content in mm^3^ (volume/ICC; mean ± SD) between healthy controls (HC) and individuals at the first-episode psychosis (FEP) from the ROIs in the left and right hemisphere attention networks. MBelt, Medial belt; STSdp, dorsal posterior superior temporal sulcus; TPOJ1, temporo-parieto-occipital junction 1; TPOJ2, temporo-parieto-occipital junction 2; V3B, visual area 3b; p10p, posterior frontal pole area 10; MST, medial superior temporal area; LO1, lateral occipital area 1; V7, visual area 7; p9-46v, posterior area 9-ventral area 46.

There were no significant correlations between PAC and gray matter in left A1 (*p*’s > 0.1) or between PLV and gray matter in the network regions (*p*’s > 0.1). Likewise, there were no significant correlations with symptoms.

### Brief discussion

FEP had significantly thinner cortices within the left hemisphere attention network. This identifies a left hemisphere network important for attention modulation with both functional and structural deficits in FEP, while the right hemisphere network only showed functional deficits.

## General discussion

In HC, auditory attention increased activity during the M100 time-window in the dorsolateral prefrontal, parietal, and precuneus areas, corresponding to canonical executive attention network areas (14, 16), with strong effects in the precuneus, a core component of attentional control (79–82). However, a direct role in the enhancement of auditory cortex M100 could not be determined without examining network dynamics.

The low-frequency executive signal was determined *via* PAC analysis, that demonstrated every auditory area, except for right LBelt, experienced increased coupling between gamma amplitude and theta phase, with a significantly increased coupling in left A1 with attention. This provides strong evidence theta frequency is the low-frequency carrier that modulates auditory regions *via* attention, consistent with previous studies (24, 25, 40, 87, 88). The right LBelt experienced maximal coupling between gamma amplitude and alpha phase. In addition, there was a potential difference between FEP with a schizophrenia diagnosis and FEP without a schizophrenia diagnosis in PAC in the right LBelt. Other attention tasks have been shown to increase alpha-gamma coupling in sensory areas, and in general, gamma power bursts are phase locked to traveling alpha waves across cortex (22, 23). Future research can investigate these differences in right LBelt further. There were no significant PAC group differences, suggesting the mechanisms underlying PAC are relatively intact in early psychosis (26, 40).

Analysis of whole brain theta coupling using the precuneus seed regions identified two unilateral cortical attention networks. A left hemisphere network included left auditory cortex, temporo-parieto-occipital junction, superior occipital, and posterior frontal pole. A right hemisphere network included right prefrontal cortex and lateral occipital cortex. FEP were significantly impaired in the ability to enhance theta connectivity between these regions in the service of attention.

These networks shared some correspondence but also had divergence with canonical auditory attention networks. The precuneus, medial belt, and temporo-parieto-occipital junction have been implicated in auditory attention (14, 16, 79, 80, 89, 90). Dorsolateral prefrontal cortex and frontal pole are key components of canonical networks for cognitive control (13, 91–93). However, several regions identified were in occipital cortex. This may be related to the cross-modal nature of the task, as these regions are implicated in visual attention modulation (89, 94–96). Thus, the precuneus may increase connectivity with these regions while attending to auditory stimuli (and ignoring visual stimuli) to downmodulate local neuronal activity within these regions. Future studies can investigate this more directly.

FEP had thinner cortices within the left hemisphere attention network. Gray matter deficits in the left hemisphere are prevalent early in the disorder, though not always identified (97–101), and correlate with positive symptoms and neurophysiological measures (64, 66, 102–105). Gray matter loss in schizophrenia is likely due to reduced neuropil (106), hypothesized to reflect dendritic regression and reduced synaptic connectivity (66, 107, 108). We speculate that neuropil reduction within this left attention network reduces synaptic connectivity with incoming network signals controlling attention-related theta increases. However, there were no direct correlations with theta connectivity increases. There may be a mediator between the two measures, a nonlinear relationship in which gray matter thins to a threshold resulting in functional deficits and further thinning does not indicate additional deficits, or no relationship at all. Future work with larger samples may further clarify this, and ongoing longitudinal scans, where progressive gray matter loss is presumed to occur, may also provide clarification.

This study had limitations. The sample size is relatively small, and the findings need replication. Subcortical deficits exist in psychosis. However, the HCP-MMP is restricted to cortex and the reliability of MEG for subcortical structures needs further verification. Future studies can use volumetric approaches to examine subcortical contributions to these deficits. Finally, it is unknown if the identified networks are generalizable to other auditory attention tasks. Visual regions identified suggest specificity to the cross-modal aspect that may be less involved in a unimodal task (e.g., attend different auditory streams).

These novel findings indicate a distributed auditory attention circuit with functional and structural deficits in very early psychosis. This circuitopathy provides a systems-level target for novel interventions, such as non-invasive brain stimulation coupled with cognitive enhancement therapy, which can be neuroprotective in early psychosis (109). Improvement of attention modulation deficits in FEP should improve real-world functioning and might improve functional outcome if targeted early in the disorder.

### Conclusion

The ability to emphasize certain stimuli, here assessed with the enhancement of auditory M100 by attention, is related to a broad distributed attentional network that utilized theta-band synchrony to modulate sensory-related gamma activity. FEP individuals show intact phase-amplitude coupling between theta and gamma in auditory cortices but show theta-band abnormalities in the extra-auditory attentional network. Whereas psychiatrically well individuals increased theta coupling within the attention network when attending, FEP did not. This suggests that the higher-order executive signal is not arriving in auditory cortices. Further, the executive areas showed gray matter thinning in left, but not right, hemisphere, although the degree of thinning did not correlate with the severity of failure to modulate theta phase-locking. Identifying the nodes of the network underlying this attentional enhancement deficit provide novel targets for selective brain stimulation treatments.

## Data availability statement

The original contributions presented in the study are included in the article/Supplementary material, further inquiries can be directed to the corresponding author.

## Ethics statement

The work described was carried out in accordance with The Code of Ethics of the World Medical Association (Declaration of Helsinki) for experiments involving humans.

## Author contributions

DS designed the study, interpreted findings, and contributed to the critical revision of the manuscript. MC and BC helped collect data. MC wrote the first draft of the manuscript. MC performed analyses with the aid and instruction of DS, BC, and AS. All authors contributed to the article and approved the submitted version.

## Funding

This work was supported by NIMH R01 MH108568 (DS), RO1 MH113533 (DS), and F31 MH119718 (MC). The NIH played no role in the collection or analysis of data or in the preparation of this manuscript. We thank the faculty and staff of the WPH Psychosis Recruitment and Assessment Core, the Conte Center for Translational Mental Health Research (P50 MH103204, David Lewis, Director), and the University of Pittsburgh Clinical Translational Science Institute (UL1 RR024153, Steven E. Reis) for their assistance in recruitment, diagnostic and psychopathological assessments, and neuropsychological evaluations.

## Conflict of interest

The authors declare that the research was conducted in the absence of any commercial or financial relationships that could be construed as a potential conflict of interest.

## Publisher’s note

All claims expressed in this article are solely those of the authors and do not necessarily represent those of their affiliated organizations, or those of the publisher, the editors and the reviewers. Any product that may be evaluated in this article, or claim that may be made by its manufacturer, is not guaranteed or endorsed by the publisher.

## Footnotes

^1^ http://imaging.mrc-cbu.cam.ac.uk/meg/Maxfilter_V2.2

^2^ https://github.com/Washington-University/HCPpipelines

^3^ https://wiki.humanconnectome.org/display/PublicData/HCP+Users+FAQ#HCPUsersFAQ-9.HowdoImapdatabetweenFreeSurferandHCP?
