# Supplementary material for: Functional connectivity and gray matter deficits within the auditory attention circuit in first-episode psychosis
Source: Front Psychiatry. 2023 Feb 13;14:1114703. doi: 10.3389/fpsyt.2023.1114703 (PMC9968732; doi:10.3389/fpsyt.2023.1114703)
Supplement: Supplementary file 1 [file Data_Sheet_1.docx]

**Supplementary materials to “****Functional Connectivity and Gray Matter Deficits within the Auditory Attention Circuit in First-Episode Psychosis”**

**Supplemental Table S1. Phase-Amplitude Coupling Changes with Attention in Psychosis Subgroups.** Changes in phase-amplitude coupling in auditory cortex regions with attention (Attend-Ignore) in individuals at the first episode of psychosis with a Schizophrenia diagnosis (Sz) and individuals at the first episode of psychosis with a non-schizophrenia diagnosis (Non-Sz FEP).

|  | Mean±SD | |  |  |
| --- | --- | --- | --- | --- |
|  | Sz (n=17) | Non-Sz FEP (n=10) | *t* | *p* |
| Left Hemisphere |  |  |  |  |
| A1 | 2.5x10^-6^ ± 3.4x10^-6^ | 3.0x10^-6^ ± 6.1x10^-6^ | -0.25 | 0.80 |
| LBelt | 1.5x10^-6^ ± 4.2x10^-6^ | 2.2x10^-6^ ± 6.2x10^-6^ | -0.36 | 0.72 |
| PBelt | 2.4x10^-6^ ± 6.4x10^-6^ | 2.8x10^-6^ ± 9.8x10^-6^ | -0.10 | 0.92 |
| Right Hemisphere |  |  |  |  |
| A1 | 5.1x10^-7^ ± 3.8x10^-6^ | 2.5x10^-6^ ± 8.0x10^-6^ | -0.89 | 0.38 |
| LBelt | -1.1x10^-7^ ± 1.7x10^-6^ | 1.4x10^-6^ ± 1.9x10^-6^ | -2.15 | 0.04 |
| PBelt | 8.8x10^-7^ ± 4.5x10^-6^ | 3.4x10^-6^ ± 7.2x10^-6^ | -1.14 | 0.27 |

*Abbreviations*: A1, primary auditory cortex; LBelt, lateral belt; PBelt, parabelt.

**Supplemental Table S2. Connectivity Changes with Attention in Psychosis Subgroups.** Changes in theta band phase-locking value with the precuneus cluster with attention (Attend-Ignore) in individuals at the first episode of psychosis with a Schizophrenia diagnosis (Sz) and individuals at the first episode of psychosis with a non-schizophrenia diagnosis (Non-Sz FEP).

|  | Mean±SD | |  |  |
| --- | --- | --- | --- | --- |
|  | Sz (n=17) | Non-Sz FEP (n=10) | *t* | *p* |
| Left Hemisphere |  |  |  |  |
| MBelt | -0.0042 ± 0.017 | 0.0036 ± 0.012 | -1.25 | 0.22 |
| STSdp | -0.0039 ± 0.019 | 0.0029 ± 0.014 | -0.99 | 0.33 |
| TPOJ1 | -0.0004 ± 0.015 | 0.0026 ± 0.012 | -0.54 | 0.60 |
| TPOJ2 | 0.0029 ± 0.018 | 0.0032 ± 0.012 | -0.05 | 0.96 |
| V3B | 0.0027 ± 0.018 | 0.0073 ± 0.020 | -0.62 | 0.54 |
| p10p | 0.0081 ± 0.015 | 0.0020 ± 0.014 | 1.04 | 0.31 |
| Right Hemisphere |  |  |  |  |
| MST | 0.0015 ± 0.018 | 0.0045 ± 0.013 | -0.47 | 0.65 |
| LO1 | -0.0016 ± 0.016 | 0.0017 ± 0.016 | -0.52 | 0.61 |
| V7 | 0.0062 ± 0.016 | 0.0012 ± 0.014 | 0.83 | 0.41 |
| p9-46v | 0.3 x10^-4^ ± 0.013 | -0.0004 ± 0.009 | 0.09 | 0.93 |

*Abbreviations*: MBelt, Medial belt; STSdp, dorsal posterior superior temporal sulcus; TPOJ1, temporo-parieto-occipital junction 1; TPOJ2, temporo-parieto-occipital junction 2; V3B, visual area 3b; p10p, posterior frontal pole area 10; MST, medial superior temporal area; LO1, lateral occipital area 1; V7, visual area 7; p9-46v, posterior area 9-ventral area 46
